# The LINCE Project: A Pathway for Diagnosing NCL2 Disease

**DOI:** 10.3389/fped.2022.876688

**Published:** 2022-03-29

**Authors:** Daniel Rodrigues, Maria José de Castro, Pablo Crujeiras, Anna Duat-Rodriguez, Ana Victoria Marco, Mireia del Toro, María L. Couce, Cristóbal Colón

**Affiliations:** ^1^Congenital Metabolic Diseases Unit, Department of Neonatology, University Clinical Hospital of Santiago de Compostela, Instituto de Investigación Sanitaria de Santiago (IDIS), European Reference Network for Hereditary Metabolic Disorders (MetabERN), Centro de Investigación Biomédica en Red Enfermedades Raras (CIBERER), Santiago de Compostela, Spain; ^2^Department of Pediatrics, Faculty of Medicine, University of Santiago de Compostela, Santiago de Compostela, Spain; ^3^Department of Neuropediatrics, Niño Jesús Children's Hospital, Madrid, Spain; ^4^Genomics Unit, La Fe University and Polytechnic Hospital, Valencia, Spain; ^5^Pediatric Neurology Unit, Vall D'Hebron University Hospital, Barcelona, Spain

**Keywords:** NCL2 disease, dried blood spot, early diagnosis, enzymatic activity, screening tripeptidyl peptidase 1

## Abstract

**Introduction:**

Neuronal Ceroid Lipofuscinosis (NCL) comprises a clinically and genetically heterogeneous group of 13 neurodegenerative lysosomal storage disorders. Neuronal Ceroid lipofuscinosis type 2 disease (NCL2), caused by the deficient lysosomal enzyme tripeptidyl peptidase 1 (TPP1), is the only one with an approved enzyme replacement treatment (ERT). Early initiation of ERT appears to modify significantly the natural history of the disease. We aimed to shorten the time to diagnosis of NCL2.

**Methods:**

In March 2017, we started per first time in Spain a selective screening program, the LINCE project, in pediatric patients with clinical symptoms compatible with NCL2 disease. The program covered the whole country. We distributed kits to pediatricians with the necessary material to assess patients. All samples in this study were received within one week of collection. Enzymatic activity determined on dried blood spots was the main method used to screen for TPP1 and palmitoyl protein thioesterase 1 (PPT1) for the differential diagnosis with neuronal ceroid lipofuscinosis type 1 (NCL1).

**Results:**

Over a period of three years, we received 71 samples. The analysis was minimally invasive, relatively cheap and fast-executing. Three cases identified as a direct result of the selective screening strategy were confirmed by genetic study of NCL2 disease with a median age of 4.5 years. Our screening method has a specificity of 100%, and, with the absence to date of false negatives. We did not detect any NCL1-positive cases.

**Conclusions:**

LINCE proved to be a simple, useful, and reliable tool for the diagnosis of NCL2, enabling clinicians to diagnose NCL2 faster. The presence of NCL2-positive cases in our population and availability of treatment may facilitate the inclusion of NCL2 in neonatal screening programs for early diagnosis.

## Introduction

Neuronal Ceroid Lipofuscinoses (NCLs, otherwise known as Batten disease) comprise a group of progressive encephalopathies that typically present during childhood and are characterized by intralysosomal accumulation of an autofluorescent lipopigment, lipofuscin, in various tissues. All except one (NCL4B) have an autosomal recessive inheritance pattern. Although NCLs are the most common cause of neurodegeneration during infancy and childhood, they are rare even as a combined group.

Since they were first described in 1903 by the British pediatrician and neurologist Frederick Batten, NCLs were originally classified according to the age of onset of clinical symptoms into four categories: infantile (NCL1), late infantile (NCL2), juvenile (NCL3), and adult (NCL4) (1).

NCLs are currently classified numerically according to their gene defect, and 13 different forms have been described (Table 1), with an incidence that varies between 0.6 and 14/100,000 newborns in different populations (2). Most forms are characterized by progressive motor and intellectual deterioration, seizures, blindness, and early death.

**Table 1:** Classification of neuronal ceroid lipofuscinoses (according to the MIM database).

**Disease**	**Phenotype MIM**	**Gene**	**Location**	**Inheritance**	**Protein product**	**Age at onset**
NCL1	256730	*PPT1*	1p34.2	AR	**PPT1 (soluble lysosomal protein)**	Variable (Infantile)
NCL2	204500	*TPP1*	11p15.4	AR	**TPP1 (soluble lysosomal protein)**	Variable (Late Infantile)
NCL3	204200	*CLN3*	16p12.1	AR	Lysosomal membrane protein	Juvenile
NCL4A	204300	*CLN6*	15q23	AR	ER membrane protein	Adult
NCL4B	162350	*DNAJC5*	20q13.33	AD	Cytosolic, associated with vesicular membranes	Adult
NCL5	256731	*CLN5*	13q22.3	AR	Soluble lysosomal protein	Variable (Late Infantile)
NCL6	601780	*CLN6*	15q23	AR	ER membrane protein	Variable (Late Infantile)
NCL7	610951	*MFSD8*	4q28.2	AR	Lysosomal membrane protein	Variable (Late Infantile/Juvenile)
NCL8	600143	*CLN8*	8p23.3	AR	ER membrane protein	Variable (Late Infantile/Juvenile)
	610003^*^					
NCL10	610127	*CTSD*	11p15.5	AR	**CtsD (soluble lysosomal protein)**	Congenital
NCL11	614706	*GRN*	17q21.31	AR	Soluble lysosomal protein	Adult
NCL13	615362	*CTSF*	11q13.2	AR	**CtsF (soluble lysosomal protein)**	Adult
NCL14	611726	*KCTD7*	7q11.21	AR	Cytosolic, partially associated with membranes	Infantile

*Available enzyme assays are shown in bold*.

* *Northern epilepsy variant; NCL9 (609055)—not molecularly characterized*.

*PPT1, palmitoyl protein thioesterase 1; TPP1, tripeptidyl peptidase 1; Cts, cathepsin; AR, autosomic recessive; AD, autosomic dominant; ER, endoplasmic reticulum*.

NCL2 (Jansky-Bielschowsky disease; MIM #204500) is one of the most frequent NCLs and is caused by autosomal recessive mutations in the TPP1 gene resulting in a deficiency of the lysosomal enzyme tripeptidyl peptidase 1 (TPP1). Clinical manifestations start between 2 and 4 years of age, with epilepsy that becomes resistant to multiple antiepileptic drugs, myoclonic ataxia, pyramidal signs, and developmental regression (3–5). The disease progresses rapidly in most patients between the ages of ~3 and 6 years and in very quick successive stages after the onset of symptoms. Life expectancy does not go beyond adolescence. Biochemically, a defect in TPP1 (6–8), a pepstatin-insensitive protease (9) leads to an accumulation of undegraded lipofuscin causing massive neuronal cell atrophy and death (10) and resulting in brain and retinal degeneration. Historically, diagnosis of NCLs was based on clinical features and the presence of ultrastructural lysosomal inclusions of various types (3, 11, 12). Nowadays, diagnosis relies on biochemical and molecular analysis.

Prompt diagnosis is critical for optimal disease management and appropriate genetic counseling because of rapid disease progression after the onset of the first symptoms (13, 14). However, early detection is very challenging owing to poor awareness of the disease and the unspecific nature of the initial symptoms, which include language delay, motor difficulties, and epilepsy (15, 16), which can be associated with other diseases such as mucopolysaccharidoses, gangliosidoses, mucolipidoses, peroxisomal disorders, mitochondrial disorders and leukodystrophies (13). In fact, the delay between the onset of symptoms and the definitive diagnosis may span from 2 to 3 years (16).

Medical management has relied on symptomatic treatment and supportive and palliative care. Fortunately, since 2017, intraventricular enzyme replacement therapy (ERT) with cerliponase alfa has been approved in the USA by the Food and Drug Administration and in Europe by the European Medical Agency for the treatment of NCL2 (17–19). Early administration of cerliponase alfa has led to a significant reduction in the rate of decline of motor and language functions (20), thus delaying disease progression. Prompt diagnosis is mandatory prior to irreversible brain damage. Here, we present a screening strategy to facilitate rapid and reliable diagnosis of NCL2.

## Methods

### Study Design

In March 2017, the Unit for the Diagnosis and Treatment of Congenital Metabolic Diseases at our Center started the LINCE project with the endorsement of the Spanish Federation for Rare Diseases (FEDER) and the Spanish Society of Pediatric Neurology (SENEP) to identify possible cases of NCL2 throughout Spain. This study has been approved by the Territorial Research Ethics Committee with the register number 2017/185.

LINCE is a selective screening program aimed at pediatric patients (0–15 years) with clinical signs and symptoms compatible with NCL2 disease. Specific kits were designed with the necessary material, as follows: WhatmanTM 903 analytical paper for the collection of dried blood spots (DBS), an informed consent form to be signed by the parents of the participating children, a guide with indications on how to collect the samples, a contact telephone number, a contact e-mail address, and a clinical guide showing the signs and symptoms to be considered (Supplementary Figure 1).

**Figure 1:**
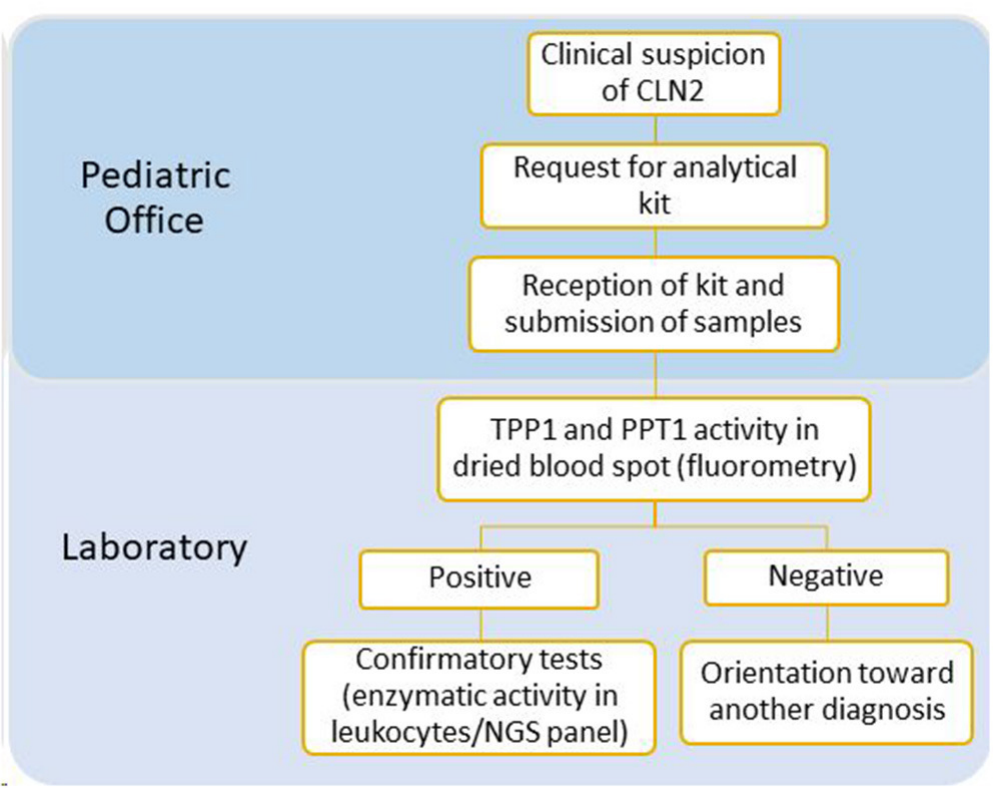
Diagnostic algorithm of the LINCE project.

Pediatricians interested in participating contacted us via email in order to obtain the LINCE kit. After reception, the enzymatic activities of TPP1 and palmitoyl protein thioesterase 1 (PPT1) were measured (Figure 1).

### Phenotype

This nationwide selective screening project targeted an at-risk pediatric population, defined as patients whose pediatricians observed clinical signs and/or symptoms compatible with a diagnosis of NCL2. The alert to the main symptoms was taken into account, including: early language delay, other developmental delays as motor difficulties or ataxia; seizures including generalized tonic-clonic, absence, myoclonic, atonic, clonic and tonic, febrile seizures; myoclonic seizures, both epileptic and non-epileptic; dramatic loss of previously attained skills as loss of voluntary movements and ability to walk and language regression; movement disorders such as myoclonus, spasticity, dystonia and chorea; visual deterioration, blindness. Samples were received with a brief description of the patient's symptoms. All probands were phenotyped using human phenotype ontology (HPO) terms extracted from the records of the medical history sent.

### Reagents

4-Methylumbelliferone sodium salt (4-MU), 7-amino-4-methylcoumarin (4-MC), 4-MU-β-D-galactoside, dimethyl sulfoxide, Triton X-100, DL-dithiothreitol, sodium azide, β-glucosidase, pepstatin A, E64 (trans-epoxysuccinyl-L-leucylamido (4-guanidino) butane), and ethylenediamine were purchased from Sigma-Aldrich Corp. (St. Louis, MO. USA). 4-MU-6-thio-palmitate-β-D-glucopyranoside was purchased from Carbosynth (United Kingdom). Ala-Ala-Phe-7-amido-4-methylcoumarin was purchased from Bachem (United Kingdom). Sodium carbonate, glycine, sodium hydroxide, acetic acid, sodium acetate, citric acid, sodium phosphate, sodium chloride, chloroform, and methanol were from Merck (Darmstadt, Germany). Bovine serum albumin (BSA) was purchased from ICN Biomedicals (Aurora, OH, USA). Ethylenediaminetetraacetic acid (EDTA) was purchased from Panreac (Barcelona).

### Enzymatic Tests

As main screening method, enzymatic activity was determined on DBS for TPP1 (EC 3.4.14.9) and PPT1 (EC 3.1.2.22) for the differential diagnosis with NCL1. Beta-galactosidase (EC 3.2.1.23) was also measured as a sample quality control. Metabolomics has demonstrated that DBS are stable where collected and transported within 28 days at room temperature (21). All samples in this study were received within one week of collection.

We adapted the methods of van Diggelen et al. (22, 23) and Ho and O'Brien (24) to evaluate the enzymatic activity of PPT1, TPP1, and β-galactosidase, respectively. Briefly, for the TPP1 measurement in DBS, a 3.2-mm punch was incubated for 20 h at 37°C with 40 μL of substrate comprising Ala-Ala-Phe-7-amido-4-MC 0.5 mM and 20 μL of sodium chloride 0.85%. The reaction was stopped with 200 μL of ethylenediamine. Substrate was added to the blanks after addition of the stopping buffer. For the PPT1 measurement, a 3.2-mm punch was incubated for 5 h at 37°C with 40 μL of substrate 4-MU-6-thio-palmitate-β-D-glucopyranoside 0.64 mM. The reaction was stopped with 300 μL of ethylenediamine.

For the biochemical confirmatory test, leukocytes were isolated from blood in EDTA tubes using the Wizard® Genomic DNA Purification Kit (Promega) and stored at −20°C until use. The leukocyte samples were diluted in 0.9 % sodium chloride solution and sonicated in an Ultrasonic Sonicator Processor (Bandelin Sonopuls HD 2070). The Bradford method was used to quantify protein in leukocytes (25). For the TPP1 measurement, 10 μg of protein was incubated for 2 h at 37°C with 20 μL of substrate comprising Ala-Ala-Phe-7-amido-4-MC 0.5 mM and 40 μL of 0.425% sodium chloride solution. The reaction was stopped with 300 μL of ethylenediamine. In the case of PPT1, 15 μg of protein was incubated for 1 h at 37°C with 20 μL of substrate 4-MU-6-thio-palmitate-β-D-glucopyranoside 0.64 mM. The reaction was stopped with 300 μL of ethylenediamine.

Fluorescence (excitation, 355 nm; emission, 460 nm) was measured on a BMG Labtech spectrofluorometer (FluoStar Optima). The readings were corrected for blanks and compared with 4-MU calibrators in the case of PPT1 and β-galactosidase, and with 7-amino-4-MC calibrators for TPP1. Enzyme activities were expressed in micromoles of 4-MU or 4-MC of product formed per h/L of blood (DBS samples) or nanomole per h/mg of protein (leukocytes) for PPT1 and TPP1, respectively.

## Results

A total of 143 kits were distributed between March 1, 2017 and May 1, 2020. Samples from 71 patients (age range: 2.5 months−15 years, 27 females, and 44 males) were received from 21 of the 17 regions of Spain. Madrid accounted for 24.4% and Valencia 13.4%. It is a minimally invasive test with a total cost per sample of around €50, compared to €80 to €100 for leukocytes and €300 for fibroblasts.

The main manifestations reported were language delay (15.5%), psychomotor delay (13.5%), and epilepsy (13.5%) (Table 2).

**Table 2:** Clinical signs reported from patients with suspected NCL2.

**Signs and symptoms**	**Number**	**%**
Language delay	24	15.5
Psychomotor delay	21	13.5
Epilepsy (refractory)	21 (8)	13.5
Myoclonic epilepsy	9	5.8
Myoclonic seizure	8	5.2
Regression	8	5.2
Visual deficit	8	5.2
Gait alteration/absence	8	5.2
Ataxia	7	4.5
Seizures	6	3.9
Encephalopathy	6	3.9
Cerebellar atrophy	4	2.6
Microcephaly	4	2.6
Hypotonia	4	2.6
Spasticity	3	1.9
Cortical atrophy	2	1.3
Aggressiveness	2	1.3
Stereotypies	1	0.6

Analysis of the DBS enzyme enabled identification of three patients with absent activity in TPP1 but normal PPT1 activity (Supplementary Table 1; Table 3). In patient 3, we also confirmed leukocyte deficiency. All cases were confirmed by genetic analysis, and three known pathogenic variants were identified (Table 3); variant c.622C>T was present in all three patients, in one patient in homozygosity, and in two patients in compound heterozygosity with variants c.509-1G>C and c.1094G>A, respectively. We only had samples from both parents for patient 3 and were able to confirm the inheritance pattern in trans in the TPP1 gene. These three cases identified as a direct result of the selective screening strategy were counted as true positives. In all samples, DBS and/or leukocytes, determination of β-galactosidase revealed normal values (data not shown).

**Table 3:** Characterization of CLN2 patients diagnosed in the LINCE project.

	**Age at diagnosis**	**TPP1 DBS (μmol/L/h) (2.0–11.0)**	**PPT1 DBS (μmol/L/h) (5.4–40.1)**	**TPP1 leukocytes (nmol/h/mg)** **(24–68)**	**PPT1 leukocytes (nmol/h/mg) (1.9–25.4)**	**CLN2 Genotype**	**Clinical signs reported at diagnosis**
Patient 1	4 years 6 months	Undetectable	9.1	Nd	Nd	c.622C>T/ c.622C>T	Delayed speech and language development Mental deterioration and motor deterioration Cerebellar atrophy by MRI Ataxia Epilepsy
Patient 2	5 years 6 months	Undetectable	13.2	Nd	Nd	c.509-1G>C/c.622C>T	Loss of speech Loss of gait Epileptic crisis
Patient 3	5 years	Undetectable	8.8	Undetectable	27.8	c.622C>T/c.1094G>A	Cognitive and motor decline Cerebellar atrophy by MRI Ataxia Epilepsy

All patients had similar ages at diagnosis (Table 3), ranging from four to five years, and shared common features such as epileptic seizures and motor decline. Ataxia and language decline were also recorded in two patients.

Patient 1, a male whose symptoms first manifested at 12 months of age as language delay followed by full stagnation of development at 15 months and cognitive deterioration at four years of age, as reported by the parents. His motor development was reported as normal. He experienced his first seizure at 3.5 years, followed by refractory epilepsy at age five despite treatment with valproate, clobazam, and levetiracetam. He presented tonic, atonic, tonic-clonic, and myoclonic seizures, together with myoclonic and atonic absences. He showed myoclonus (3.5 years), spasticity, and dystonia (4–4.5 years) with motor regression and total loss of gait at the age of five. Visual impairment was observed at 3.5 years, progressing to total blindness at five. Intraventricular ERT was not started after the diagnosis, as the child did not meet the inclusion criteria because of the severity of his disease. He died at the age of 6 years old.

Patient 2, a female whose symptoms first manifested at 12 months of age as language delay followed by cognitive deterioration starting at 2.5 years of age and coinciding with the first seizures. She experienced absences and right focal tonic-clonic seizures, requiring three antiepileptic drugs due to refractory epilepsy. She also had axial tremor, myoclonus, oro-facial dyskinesias, and oculomotor apraxia. No visual deficit was recorded. ERT was initiated at the age of diagnosis (five years and six months), although it was interrupted after six months due to lack of response and deterioration.

Patient 3, a male who showed the first symptoms of the disease at 3.5 years of age, namely, mild language delay and clumsiness. Cognitive decline was observed at four years of age, coinciding with the onset of epilepsy. He experienced his first seizure at three years and 11 months of age, followed by refractory epilepsy at age five. He currently requires treatment with three anticonvulsants: valproate, clobazam, and levetiracetam. His seizures are tonic, atonic, tonic-clonic, and myoclonic, together with absences. He also has myoclonus and spasticity, together with ataxia, tremor, and choreic movements of the upper extremities. He has needed a wheelchair since the age of five years, although he is able to crawl. Visual deficit has not been evidenced. Intraventricular ERT was started at the age of diagnosis (five years). He remains stable after one year on therapy. Currently, at six years of age he has a comprehensive language, and although expressive language is mostly unintelligible, he tries to communicate and says some words with meaning.

## Discussion

Clinician awareness and regional differences in availability of TPP1 testing may affect prompt diagnosis. Furthermore, ensuring that clinicians are aware of NCL2 during the differential diagnosis may facilitate early treatment with ERT. LINCE is an acronym for Spanish Neuronal Ceroidea Lipofuscinosis. Our goal is not only to speed up the diagnosis of NCL2 disease for physicians who suspect a NCL, but also to create awareness of these dramatic, debilitating diseases. In this study, we demonstrated that diagnosis of NCL2 is possible using a simple, selective screening method. The use of DBS as a first-tier sample is very convenient, since it is easy to collect and remains stable at room temperature; therefore, it can be sent by ordinary mail (26–28). It is also very cost-effective compared with leukocyte or fibroblast samples (13, 29), which can be particularly sensitive to temperature variations and shipping delays, thus ultimately reducing assay reliability (13). The diagnostic method is based on fluorometric enzyme analysis for both DBS and leukocyte samples, and no residual activity was found in any sample from NCL2 patients. No false-negative results have been reported to date, and no NCL1 patients have been identified. More recently, tandem mass spectrometry (MSMS) assays for the determination of TPP1, PPT1 and cathepsin D activity in DBS have become available (30–34). Although MSMS assays yield higher analytical ranges than fluorometric assays (34), one of the advantages of our method is that it can measure PPT1 in only 5 h and TPP1 in 20 h, as opposed to the 45-h incubation reported by Lukacs et al. (26) and similar to LC-MS/MS multiplex assays (34). Efforts are currently being made in our laboratory to measure both TPP1 and PPT1 in the shortest time possible using mass spectrometry, because are the most commons.

Unfortunately, since NCL2 and NCL1 are among the few NCLs with lysosomal soluble proteins that can be measured to support a diagnosis, genetic analysis is the only diagnostic option for the remaining NCLs. Two of the patients in the present series were compound heterozygous, the first was homozygous, and the variants had already been described and classified as pathogenic (35). Of the 131 known NCL2 variants (34), c.509-1G>C and c.622C>T were found in all three patients. These variants are the two most frequent and account for 50% of disease-associated alleles (89% in Europe). Both variants have been associated with classic late-infantile and atypical NCL2 (8% juvenile, 3% spinocerebellar ataxia, and <1% spastic paraplegia or congenital disease), suggesting that additional genetic factors are likely to contribute to earlier disease onset. The other variant, c.1094G>A, represents 2% of disease associated alleles and may be associated with an increased probability of classic late-infantile NCL2. However, specific disease genotypes do not consistently correlate with phenotypes (36).

Several therapeutic approaches for NCLs are currently under investigation, including gene therapy, neuronal stem cell therapy, immunomodulation, small molecule therapy, and ERT (37–41), which is the only approved treatment for NCL2. Cerliponase alfa, a recombinant proenzyme form of human TPP1, was granted regulatory approval in the USA and Europe for the treatment of NCL2 disease (specifically for slowing the loss of ambulation in symptomatic children aged ≥3 years in the USA). It is administered by intraventricular infusion every 2 weeks and has demonstrated less decline in motor and language function than in historical controls. The maximum benefit from ERT can be obtained if diagnosis is made as early as possible in order to avoid the irreversible neurodegeneration often present when diagnosis is made at around five years of age, and also because the disease progresses very rapidly immediately after onset of the initial symptoms. As can be seen from the most common signs (Table 2), language delay and/or psychomotor delay are often associated with unexplained seizures, which should raise the suspicion of NCL2.

### Limitations

The study has been carried out in patients with symptoms and with voluntary participation, because of this does not reflect the real incidence.

## Conclusions

In summary, the LINCE project is a simple, useful, not very expensive, and novel tool that speeds up the diagnosis of NCL2. All positive cases experienced a rapid decline in their medical condition, which is characteristic of NCL2 patients. An early diagnosis is key to accessing treatment, but diagnosis is challenging because of similarities with other disorders and limited awareness of NCL2 disease because of its rarity. Since therapy is now available, screening for NCL2 should be implemented in neonatal screening programs for early detection, ultimately leading to better outcomes.

## Data Availability Statement

The original contributions presented in the study are included in the article/Supplementary Material, further inquiries can be directed to the corresponding authors.

## Ethics Statement

The studies involving human participants were reviewed and approved by Ethics Committee Santiago-Lugo with the register number 2017/185. Written informed consent to participate in this study was provided by the participants' legal guardian/next of kin.

## Author Contributions

CC, MJC, and MLC contributed to design of study. DR, PC, and CC contributed to analysis and interpretation of data. AD-R, AM, and MT contributed to acquisition of clinical data. All authors have participated and/or reviewing, the manuscript and have approved its submission.

## Funding

This study received funding from Grant Biomarin Inc. The funder was not involved in the study design, collection, analysis, interpretation of data, the writing of this article or the decision to submit it for publication.

## Conflict of Interest

The authors declare that the research was conducted in the absence of any commercial or financial relationships that could be construed as a potential conflict of interest.

## Publisher's Note

All claims expressed in this article are solely those of the authors and do not necessarily represent those of their affiliated organizations, or those of the publisher, the editors and the reviewers. Any product that may be evaluated in this article, or claim that may be made by its manufacturer, is not guaranteed or endorsed by the publisher.
