# Supplementary figures and images for: The LINCE Project: A Pathway for Diagnosing NCL2 Disease
Source: Front Pediatr. 2022 Mar 29;10:876688. doi: 10.3389/fped.2022.876688 (PMC9002010; doi:10.3389/fped.2022.876688)

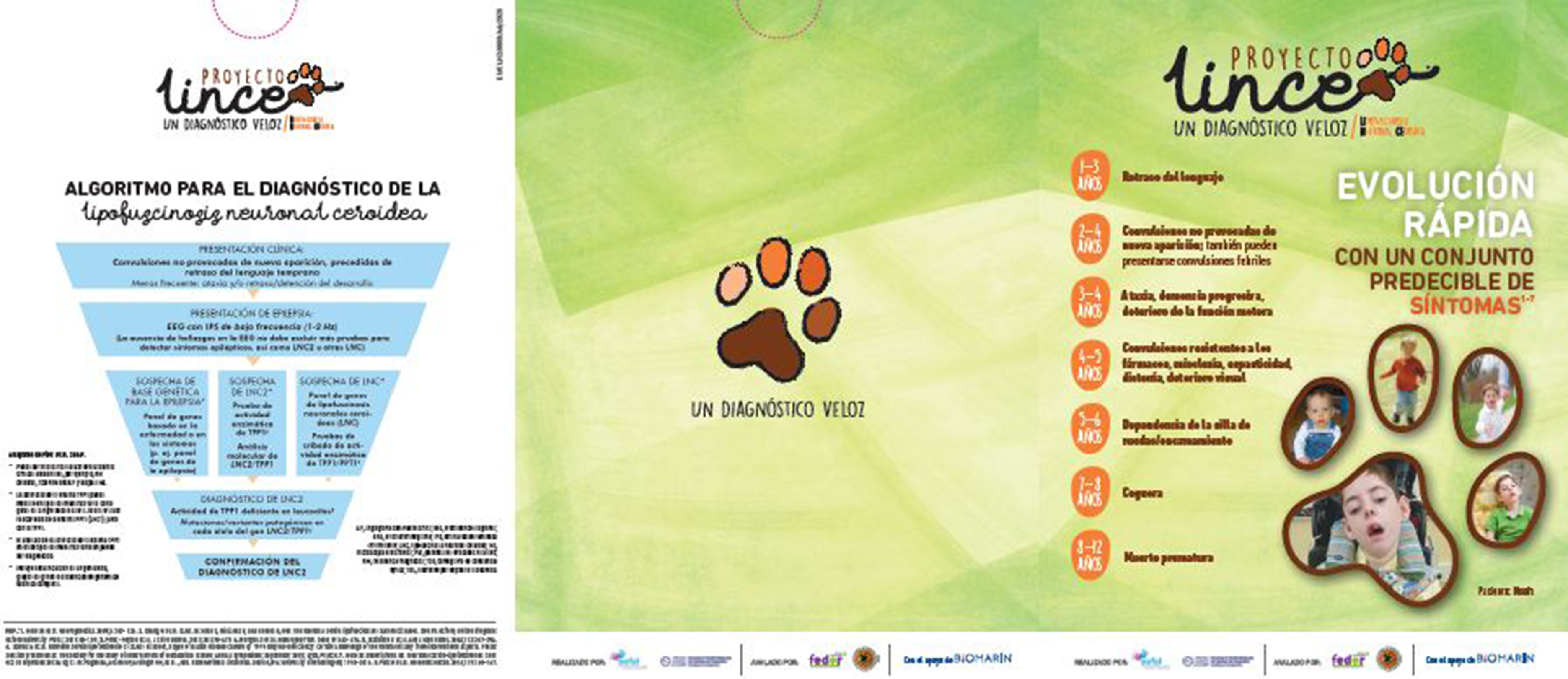

Supplement: Supplementary file 2 [file Image_1.JPEG]
